# Editorial: Current Perspectives on Insulin-Like Growth Factor Binding Protein (IGFBP) Research

**DOI:** 10.3389/fendo.2018.00667

**Published:** 2018-11-20

**Authors:** Andreas Hoeflich, John Pintar, Briony Forbes

**Affiliations:** ^1^Institute of Genome Biology, Leibniz-Institute for Farm Animal Biology (FBN), Dummerstorf, Germany; ^2^Department of Neuroscience and Cell Biology, Rutgers Robert Wood Johnson Medical School, Piscataway, NJ, United States; ^3^College of Medicine and Public Health, Flinders University, Bedford Park, SA, Australia

**Keywords:** IGFBP, growth, metabolism, cancer, aging, health, biomarker, proteases

The insulin-like growth factor binding proteins (IGFBPs), as high affinity IGF binding partners, are the principal regulators of IGF-1 and IGF-2 action. Accordingly, effects of IGFBPs can be observed on the levels of growth and differentiation, development, metabolism, and lifespan. The diversity of IGFBP-actions arises due to time-, sex-, and tissue-specific expression of the six distinct IGFBPs (IGFBP-1 to−6), which have redundant functions as seen from the analysis of double-, triple-, or quadruple IGFBP-knockout mice. The complexity of IGFBP functions is related not only to their roles as IGF carriers within the circulation but also to actions within the extracellular space and in distinct subcellular compartments, such as the cell nucleus. IGFBP functions have been attributed to structural motifs in the three conserved IGFBP subdomains, with specific residues being posttranslationally modified by glycosylation or phosphorylation to regulate IGFBP action. In addition, multiple binding partners inside and outside the cell have been identified that regulate IGFBP functions, including their IGF-independent activities. Furthermore, an in-depth understanding is emerging of the role of IGFBP proteolysis in the regulation of both IGF-dependent and IGF-independent actions through generation of potentially bioactive IGFBP-fragments. Accordingly, proteolytic degradation of IGFBPs as a physiologically relevant mechanism in disease has been revealed both in a malignant context but also in other acute or chronic pathophysiological conditions. Finally, the IGFBPs e.g., as sensors of GH/IGF-status have tremendous biomarker potential. Measurement of IGFBP-3/IGFBP-2 ratios provides ultimate sensitivity for the GH-status of a given cellular system. Similarly, detection of intact and fragmented IGFBPs may provide an indication of disease status. Accordingly, for the future we may expect an evolution of IGFBP-related diagnostic approaches, which extend to the characterization of both structural and functional properties of IGFBPs and their fragments in preclinical and clinical research.

In the present Research Topic we present both original work and reviews on the potential roles of IGFBPs in organisms ranging from fish, farmed animals (pigs and cows) to human. One of the most intriguing questions discussed by Allard and Duan is why there are so many IGFBPs. Because the different IGFBPs have evolved from one common ancestor, it is thought that the IGFBPs were used to increase functional biodiversity with respect to IGF-dependent and independent functions. This increase of diversity seems to be true particular in teleost fish where up to 4 copies may have arisen from IGFBP-2 due to genome duplications (Garcia de la Serrana and Macqueen). Evidence continues to accumulate for active and biologically significant proteolytic fragments of a number of IGFBPs. Some of this evidence is summarized in reviews on the role of IGFBP-proteases in bone (Beattie et al.) and for ovarian folliculogenesis (Mazerbourg and Monget). As mentioned above, IGFBPs may serve as biomarkers for a number of clinical conditions. However, there is need for additional structural information on the IGFBPs before this goal can be achieved, as in the example of cardiovascular diseases (Hoeflich et al.). The biomarker potential of IGFBPs was also critically discussed for autoimmune diseases because IGFBPs by IGF-dependent or -independent mechanisms also control proliferation of immune cells (Ding and Wu). Interestingly, IGFBP-5 was identified as an effector of cell senescence under the control of inflammatory signals and fibrinolysis and pro-coagulation (Sanada et al.). During pulmonary fibrosis, IGFBP-5 can increase its own expression which may increase the fibrotic effect in primary human lung fibroblasts by increasing expression of extracellular matrix proteins (Nguyen et al.). The assessment of either isolated IGFBPs or more complex IGFBP-signatures revealed novel biomarker potential for IGFBPs in different experimental systems ranging from malignant cells to farm animals. Accordingly, nuclear presence of IGFBP-3 in triple-negative breast cancer cells indicates a worse prognosis (Julovi et al.). Interestingly a link between obesity and malignant growth in prostate cancer cells was suggested as hyperglycemia was demonstrated to increase the expression of IGFBP-2 (Holly et al.). By contrast in farm animals, serum concentrations of IGFBP-2 and−3 were identified as novel biomarkers for intermediate stress conditions (Wirthgen et al.) or the maintenance of pregnancy (Mense et al.). Finally also gender aspects have to be considered since two independent contributions discussed the potential roles of IGFBPs for male (Safian et al.) and female (Spitschak and Hoeflich) reproductive development.

The different contributions *par excellence* provide substantial evidence that functional analysis of IGFBPs and IGFBP-related biomarker research contains huge potential but is only in its infancy. Future studies on the roles of IGFBPs need to take not only sex, age, and gender into consideration but also the metabolic conditions, subcellular localization, and IGFBP structural information. Taken together this would also lead to subsequent improvement in analytical method standardization.

We want to thank all authors for their fine contributions and all reviewers and editors for their critical revision of the manuscripts. Last but no least we are indebted to the Frontiers team and Emilie Schrepfer for her invaluable support with the development of the Research Topic.

## Author contributions

All authors listed have made a substantial, direct and intellectual contribution to the work, and approved it for publication.

### Conflict of interest statement

AH is related to Ligandis GbR. The remaining authors declare that the research was conducted in the absence of any commercial or financial relationships that could be construed as a potential conflict of interest.

